# Evaluating the Joint Toxicity of Two Benzophenone-Type UV Filters on the Green Alga *Chlamydomonas reinhardtii* with Response Surface Methodology

**DOI:** 10.3390/toxics6010008

**Published:** 2018-01-10

**Authors:** Feijian Mao, Yiliang He, Karina Yew-Hoong Gin

**Affiliations:** 1 Department of Civil and Environmental Engineering, National University of Singapore, 1 Engineering Drive 2, E1A 07-03, Singapore 117576, Singapore; maofeijian@u.nus.edu; 2 School of Environmental Science and Engineering, Shanghai Jiao Tong University, Shanghai 200240, China; ylhe@sjtu.edu.cn; 3 NUS Environmental Research Institute, National University of Singapore, 5A Engineering Drive 1, #02-01, Singapore 117411, Singapore

**Keywords:** benzophenone-1, benzophenone-3, *Chlamydomonas reinhardtii*, response surface methodology, joint toxicity

## Abstract

The widespread occurrence of benzophenone-type ultraviolet (UV) filter has raised the public concerns over the ecotoxicological effects of these chemicals. The present study assessed the joint toxicity of two representative benzophenones, benzophenone-1 (BP-1) and benzophenone-3 (BP-3), on the green alga *Chlamydomonas reinhardtii* using response surface methodologies (RSM). Specific growth rate and photosynthetic pigments were used as endpoints to evaluate the toxic effects. Generally, exposure to the combined BP-1 and BP-3 negatively affected cell growth and pigments production, with higher inhibitions at higher exposure concentrations. The simultaneous reduction in growth rate and pigments contents indicated that BP-1 and BP-3 regulated the growth of the tested alga by affecting the photosynthesis process. Results also showed that second order polynomial regression models fitted well with experimental results for all endpoints. The obtained regression models further indicated that the effects of the combination stemmed significantly from the linear concentration of BP-1 and BP-3. The overall results demonstrated that RSM could be a useful tool in ecotoxicological studies.

## 1. Introduction

Benzophenone (BPs), a group of ultraviolet (UV) light absorbers, are widely incorporated in various personal care products (e.g., sunscreens, body lotions, hair sprays and shampoos) [1,2]. Benzophenone-3 (BP-3), one of the most popular BP-type UV filters, has been available as an active sunscreen ingredient for more than 40 years [3]. According to the European Chemicals Agency (ECHA), BP-3 is manufactured and/or imported into the European Economic Area at approximately 100–1000 tons per year [4]. As an active ingredient in sunscreen products, BP-3 is permitted at levels of up to 5–6% in Japan, Korea and U.S.A., and up to 10% in Australia, China and Europe [5]. It was estimated that nearly 65% of chemical sunscreen products contain BP-3 [6]. In addition, BP-3 was found in over 80% of 231 personal care products collected from China and USA, at concentrations up to 1.48 mg g^−1^ [7]. To achieve better protection against UV light, other BPs are also used in sunscreen products. For example, Benzophenone-1 (BP-1), Benzophenone-2 (BP-2), BP-3 and Benzophenone-6 (BP-6) are allowed to be added into sunscreens in Japan and South Africa [5,8]. Apart from personal care products, BP-type UV filters are also used in synthetic products such as food packaging, insecticides and paints that are exposed to sunlight [1,2,9]. 

As a result of extensive usage and continuous disposal, these BP-type UV filters can reach ambient aquatic environments directly from water recreational activities (e.g., swimming) and indirectly from sewage discharges (e.g., effluent of wastewater treatment plants), making them a group of “pseudo-persistent” contaminants [2,3,10]. In recreational waters such as swimming pools, concentrations of BP-1 and BP-3 were detected up to 8700 ng L^−1^ and 4500 ng L^−1^, respectively [11]. However, concentrations may reach mg L^−1^ levels when the number of swimmers is high [12]. High concentrations (mg L^−1^ or several hundreds of µg L^−1^) of BPs were also reported in wastewater influents and effluents [13]. Following discharge, BP compounds are frequently detected in aquatic systems, with reported concentrations typically ranging from ng L^−1^ to low µg L^−1^ in water samples [3,10,14]. Consequently, aquatic organisms are exposed to a wide range in BPs, possibly resulting in toxic effects. 

Phytoplankton play an important role in aquatic ecosystems as they form the base of the food chain/web. These unicellular organisms are sensitive to environmental stresses, making them excellent candidates for indicators of water contamination and ecotoxicological studies [15,16,17]. In fact, various studies have tested the effects of BP-type UV filters on these organisms. From these studies, half maximal effective concentration (EC_50_) values of BP-3 for green algae were estimated to be 0.25 mg L^−1^ for *Skeletonema pseudocostatum*, 0.96 mg L^−1^ for *Desmodesmus subspicatus*, 1.85 mg L^−1^ for *Chlamydomonas reinhardtii* and 22.4 mg L^−1^ for *Chlorella vulgaris* [18,19,20,21]. The EC_50_ (72 h) of BP-1 was reported to be 10.5 mg L^−1^ for green alga, *Raphidocelis subcapitata* [22]. These studies suggested that BP-type UV filters can affect the multiplication of green algae individually. However, there is limited information on the joint toxicity of BPs in mixtures, which is more realistic because BPs usually occur in mixtures in aquatic environments [10,23,24].

Traditionally, the effects of contaminants in mixture have been assessed by the concentration addition (CA) model, the independent action (IA) model and combination index (CI) model [25,26]. However, these models failed to fully characterize actual changes in the tested organisms, nor the possible interactions of different tested parameters [25,27]. These disadvantages can be improved by response surface methodology (RSM). RSM was introduced in the early 1950s and aimed at optimizing industrial chemical reactions [25,28]. RSM uses a variety of mathematical algorithms and can help to obtain a holistic response affected by several variables [25,29]. Another advantage of this method is that it can graphically show the responses by a three-dimensional plot and a contour plot (Figure S1). Therefore, this methodology has recently been suggested to be a good candidate for evaluating the toxic responses affected by several environmental factors [25,27,29]. The first successful application of RSM in toxicity testing was performed to evaluate the effects of chromium on a green alga (*Pseudokirchneriella subcapitata*) [27]. This approach was further expanded but limited to other contaminants (e.g., pesticide and antibiotics) and other organisms (e.g., rotifer and common carp) [25,29,30]. To further evaluate the suitability of this method in toxicity testing, more contaminants and organisms should be included [25].

The objective of the present work therefore, was to evaluate the effects of BP-1 and BP-3 in binary combination on the green alga, *C. reinhardtii*, with RSM. These two BPs were selected based on their usage, environmental occurrence and toxicological effects. To the best of our knowledge, this is the first study evaluating the joint toxicity of two BP-type UV filters in phytoplankton.

## 2. Materials and Methods 

### 2.1. Test Substances 

BP-1 and BP-3 were purchased from Sigma-Aldrich (Sigma-Aldrich, Singapore). The Chemical Abstract Services (CAS) number, physicochemical properties are listed in Table 1. Stock solutions (5000 mg L^−1^) were prepared in methanol (HPLC grade, Sigma-Aldrich, Singapore) and stored at −20 °C in the dark.

### 2.2. Preparation of the Algae Culture

The tested green alga, *C. reinhardtii* (strain NIES 2463), was obtained from the Japanese Microbial Culture Collection at the National Institute for Environmental Studies (NIES Collection). Following the Organization of Economic Co-operation and Development (OECD) test guidelines [31], the strain was maintained in about 400 mL sterilized McBride *Listeria* agar (MLA) medium at an initial inoculation of 10% concentration (*V_inoculum_*/*V_medium_*) in several TPP tissue culture flasks (growth surface: 300 cm^2^; with filter; TPP Techno Plastic Products AG, Trasadingen, Switzerland) [32]. The strain was kept in a constant temperature room (28 ± 1 °C) under cool white fluorescent lamps with a light intensity of 25 ± 2 μmol photons m^−2^ s^−1^ (12/12 h light/dark photoperiod) [21]. To ensure homogenous cell growth, the flasks were shaken and randomly relocated three times a day to eliminate the variations in light intensity at different positions. At logarithmic growth stage (about 7 days), the cells were harvested by centrifugation (5000 *g*, 4 °C, 10 min) [21]. The cell pellets were then washed three times with sterilized MLA medium. The concentrated cell mass was stored at 4 °C in the dark for a maximum of 7 days before downstream experiments [21].

### 2.3. Experimental Design

In RSM, the Box-Behnken design (BBD) and the central composite design (CCD) are two commonly used designs [33]. The CCD model is capable of providing sufficient information on the direct effects and possible interactive effects of the tested factors [27]. Therefore, the CCD model was selected for experimental design in the present study. The CCD model consists of three portions: (1) a complete or a part of factorial design in the factors studied; (2) a set of axial points; and (3) a set of center points [28]. The CCD model in the present study is a 2^2^ factorial design, where exposure concentrations of BP-1 and BP-3 were the two independent variables that may be toxic to the tested alga. The two BPs were studied at five levels: 0, ±1 and ±*α*. Here $\alpha =\sqrt{2}$ for two tested variables [28]. The tested variables were coded following the equation bellow [27]:

*x_i_* = (*X_i_* − *X*_0_)/*ΔX_i_*, *i* = 1, 2, 3, …, *k* (1)

where *x_i_* is the coded value and *X_i_* is the actual value of the *i*th independent variable. *X*_0_ is the actual value of the center point. *ΔX_i_* is the change value in every step. The coded and actual exposure levels of BP-1 and BP-3 are shown in Table 2. The experimental design, model evaluation and significance analysis (analysis of variance, ANOVA) were performed on Design-Expert software (version 8.0.6, Stat-Ease, Minneapolis, MN, USA).

### 2.4. Toxicity Tests

The toxicity tests were performed according to OECD (2001). In brief, 1 mL of the concentrated cell mass was inoculated in a TPP flask (growth surface: 75 cm^2^; with filter) containing 50 mL MLA medium, reaching an initial cell density of approximately 1.5 × 10^5^ cells mL^−1^. Prior to inoculation, the medium was spiked with determined concentrations of BP-1 and BP-3 following the central composition design (CCD) model in RSM (Table 2). A preliminary test was performed to check the toxicity of individual BP-1 or BP-3 to *C. reinhardtii* (Figure S2). The results showed a clear dose response effect when concentrations were higher than 1 mg L^−1^. EC_50_ values of BP-1 and BP-3 were calculated to be 4.23 and 2.29 mg L^−1^, respectively. Together with the fact that BPs may reach low mg L^−1^ level in the environment, the range of 1–5 mg L^−1^ was selected as the tested concentration. In all BP-1- and BP-3-treated groups, an appropriate volume of methanol was added to the medium to make up the same final methanol concentration to 0.1% (*v*/*v*). The control experiment was performed by adding methanol to the BPs-free medium to a final concentration of 0.1% (*v*/*v*). The cultures were incubated for three days under the conditions described previously. All the experiments were performed in triplicate. Optical density (OD_680_) was monitored every day. Concentrations of three photosynthetic pigments (i.e., chlorophyll a (chl-a), chlorophyll b (chl-b) and carotenoid) were measured on the last day (day 3) of the experiments.

### 2.5. Measurement of Cell Growth and Photosynthetic Pigments

The obtained optical density (OD) values were converted to cell density based on a developed linear relationship between OD and cell density ((OD_680_ + 0.0059)/2 × 10^7^ cells mL^−1^, *R*^2^ > 0.99). The specific growth rate (µ) was then calculated by fitting the cell number to the following exponential function [34]:

µ = (*lnN*_2_ − *lnN*_1_)/(*t*_2_ − *t*_1_) (2)

where *N*_1_ and *N*_2_ are the cell numbers at time *t*_1_ and *t*_2_, respectively.

The concentration of the three pigments were measured following available methods [35]. In brief, a 5-mL culture was collected by centrifugation at 4000 rpm for 10 min and the supernatant was discarded. The cell pellet was re-suspended in 5 mL of 90% methanol. The sample was sonicated for 3 min and then kept in the dark at 4 °C for 24 h before centrifugation (4000 rpm, 4 °C, 10 min). The absorbance of the supernatant was recorded at 470, 652, 665 and 750 nm. The contents of chl-a, chl-b and catenoid were estimated by the following equations:

chl-a (µg mL^−1^) = 16.72*A*_665_ − 9.16*A*_652_ (3)

chl-b (µg mL^−1^) = 34.09*A*_652_ − 15.28*A*_665_ (4)

carotenoid (µg mL^−1^) = (1000*A*_470_ − 1.63 chl-a − 15.28 chl-b)/221 (5)

Absorbencies at 470, 652 and 665 nm were corrected by subtracting the absorbance at 750 nm (turbidity).

Growth variation (%) was assessed by comparing the specific growth rates and concentrations of pigments of the treated groups with the results for the controls. A lower variation value indicates a higher inhibition effect.

### 2.6. Measurement of BP-1 and BP-3

Initial exposure concentrations of BP-1 and BP-3 in the culture medium were measured before inoculation. To enable the detection of BP-1 and BP-3, 1 mL of the MLA medium was collected and filtered through a 0.2 µm of PTFE Syringe Filter. The filtrate was diluted by 10 times with ultrapure water. Before instrumental analysis, the diluted filtrate was spiked with 20 ng of the isotopically-labeled standard (dissolved in methanol), with a water methanol ratio of 4:1 (*v*/*v*). The target BPs were quantified by high performance liquid chromatography tandem-mass spectrometry (HPLC-MS/MS) (Dionex Ultimate 3000, Dionex, Sunnyvale, CA, USA) coupled with electro-spray ionization (ESI) tandem mass spectrometry (AB Sciex Qtrap 5500, Toronto, ON, Canada) equipped with an Agilent Poroshell 120 EC-C18 reverse phase column (100 mm length × 4.6 mm internal diameter; 2.7 μm particle size, Agilent, Palo Alto, CA, USA) connected with a guard column (20 × 2.1 mm, 5 μm; Thermo Electron Corporation, Bellefonte, PA, USA), at a flow rate of 0.4 mL min^−1^. The mobile phases were 5 mM ammonium acetate in Milli-Q water (mobile phase A) and acetonitrile/methanol (1/1, *v*/*v*) (mobile phase B). The gradient elution started with 10% B at 0 min, held for 0.5 min; linearly increased to 50% B at 0.5 to 1.5 min, then linearly increased to 95% B at 1.5 to 9 min, held for 6 min to 15 min, then re-equilibrated the column to initial conditions at 15.1 min and stabilized for 2 min. The overall run time was 17 min. The injection volume was 10 μL and the column temperature was 25 °C.

According to OECD guidelines, the variations between actual concentrations and nominal concentrations (levels coded by the CCD model for the present study) should not exceed 20%, as was the case in this study [31]. Therefore, the nominal concentrations were used for model development.

## 3. Results

**Figure 1:**
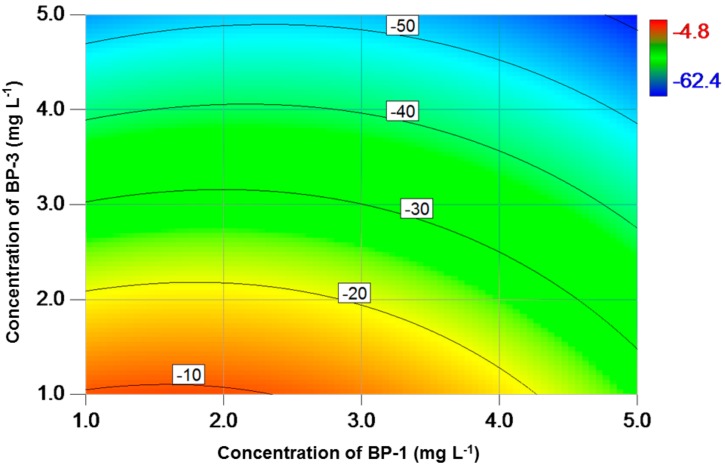
Contour plot of variation (%) in specific growth rate as a function of BP-1 and BP-3 concentrations for *C. reinhardtii*. A low variation value indicates a high inhibition effect.

The contour plot of variation in specific growth rate (µ) as a function of BPs exposure concentration is shown in Figure 1. The results suggested that the growth of alga was inhibited over the entire exposure concentration. The overall inhibition indicated that BP-1 and BP-3 were toxic to the tested alga, where the inhibition percentage increased from 5% to 62% following increased exposure level. The highest inhibition was observed when the concentrations of BP1 and BP-3 were at 5 mg L^−1^.

**Figure 2:**
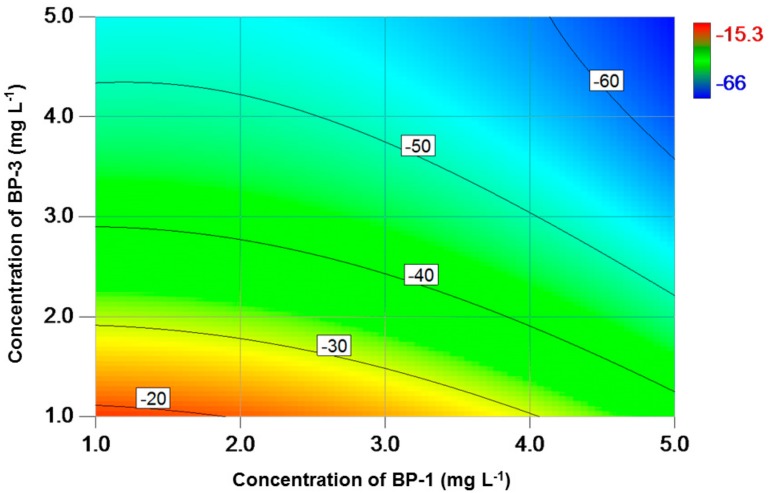
Contour plot of variation (%) in chl-a concentration as a function of BP-1 and BP-3 concentrations for *C. reinhardtii*. A low variation value indicates a high inhibition effect.

**Figure 3:**
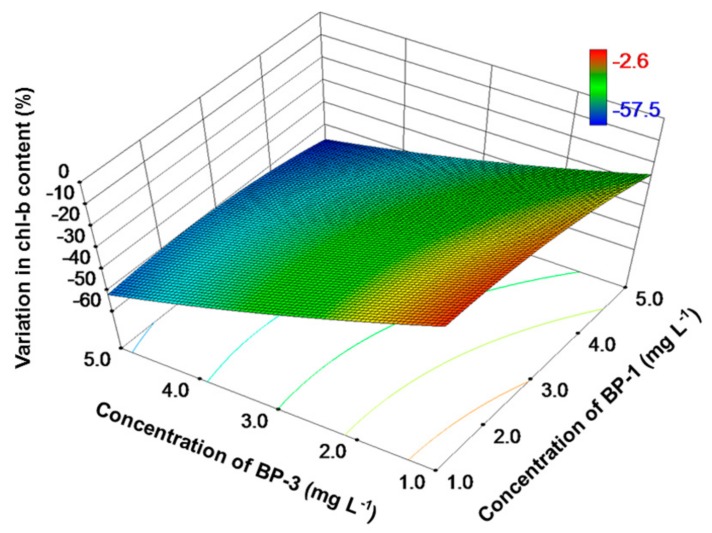
Response surface plot of variation (%) in chl-b content as a function of BP-1 and BP-3 concentrations for *C. reinhardtii*. A low variation value indicates a high inhibition effect.

**Figure 4:**
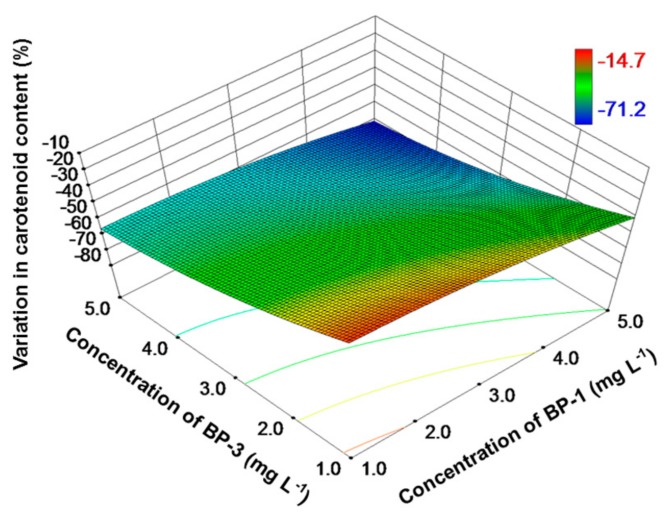
Response surface plot of variation (%) in carotenoid content as a function of BP-1 and BP-3 concentrations for *C. reinhardtii*. A low variation value indicates a high inhibition effect.

The concentrations of chl-a were also reduced over the entire exposure range, with a higher reduction rate (15–66%) when compared to specific grow rate (Figure 2). Similar to specific growth rate, the inhibition of chl-a was lowest at the two lowest exposure concentrations (1 mg L^−1^). The inhibition rate increased when the exposure concentrations increased, reaching a maximum at the two highest exposure concentrations (5 mg L^−1^). As for the remaining two pigments, that is, chl-b and carotenoid, high concentrations of BP-1 and BP-3 also caused a high inhibition (Figure 3 and Figure 4). With a relatively higher inhibition (15–71%), carotenoid was more sensitive than chl-b (inhibition rates: 3–58%) when exposed to the binary mixture of BP-1 and BP-3.

To better evaluate the toxic responses and possible interactions between the two factors, a second-order polynomial model was established to fit the experimental results for each response:

*Y* = *a*_0_ + *a*_1_*x*_1_ + *a*_2_*x*_2_ + *a*_11_*x*_1_^2^ + *a*_22_*x*_2_^2^ + *a*_12_*x*_1_*x*_2_ (6)

where *a*_0_, *a*_1_, *a*_2_, *a*_11_, *a*_22_, *a*_12_ are constant coefficients, and $x_{1}$, $x_{2}$ are the coded exposure concentrations for BP-1 and BP-3, respectively. The constant coefficients indicate the effects of exposure concentrations and the interaction between the two BPs. A synergistic effect is expected when the sign of the coefficient is positive, and an antagonistic effect is expected when the sign is negative [29]. Table 3 shows the adequacy of the fitted models as evaluated by analysis of variance (ANOVA). These results showed that the fitted models were significant (*p* < 0.05). The relatively high determination coefficients (*R*^2^ > 0.94) indicated that the models provided adequate representation the four employed endpoints. In addition, the lack of fit test for the four endpoints implied a non-significant lack of fit (*p* > 0.05), indicating that the obtained models were suitable. The statistical results also suggested that the four responses were significantly affected by the linear concentrations of the two BPs (*a*_1_, *a*_2_, *p* < 0.05). No significant combined effects were observed ($a_{12}$, *p* > 0.05, *F*-test) and only chl-a was significantly influenced by the second order of BP-3 ($a_{22}$, *p* < 0.05, *F*-test).

## 4. Discussion

In the past decade, the joint toxicity of BP-type UV filters has attracting growing scientific interest as they are generally used in combination in commercial products and usually co-occur in the aquatic systems [3]. Reported additive, antagonistic and synergistic effects of mixtures of BPs indicate a significant interaction among them [36,37,38]. For example, one study observed an additive effect of BP-1 and BP-3 in estrogen receptor (ER) binding in MCF-7 cells, potentially due to their similar mode of action [37]. In addition, synergistic effects of mixtures of BPs were observed in a recombined yeast assay with the human estrogen receptor alpha (hERα) [38]. Contrary to these studies, antagonism was reported for mixture of BPs with vitellogenesis in fish [36]. Likewise, Molins-Delgado (2016) found that co-exposure reduced the overall toxic effects of BP-1 and BP-3 to the zooplankton, *Daphnia magna*. The above-mentioned studies indicate that the mechanism of joint toxicity may depend on the tested organisms and endpoints. However, available joint toxicity tests mainly employed cell lines (e.g., MCF-7 cells and recombinant yeast), invertebrate and fish as test models, with a paucity of information on phytoplankton, one of the most important organisms in the aquatic ecosystem [36,37,38]. The novelty of the current work is, therefore, evaluating the toxicity of a binary mixture of BP1 and BP-3 to green algae, with *C. reinhardtii* as an example due to its widespread distribution and environmental relevance [39].

Consistent algal growth inhibition was observed in the present study, especially for higher BP-1 and BP-3 concentrations (Figure 1). This agrees with a previous report that BP-1 and BP-3 can cause growth inhibition of green algae in the low mg L^−1^ range [20,22]. In facts, a much lower effect concentration (72-h EC_50_: 13.9 µg L^−1^) was reported for a marine algal species, *Isochrysis galbana* [40]. It is clear that growth inhibition increased more rapidly when concentration of BP-3 increased (Figure 1). This indicates that BP-3 is more toxic to *C. reinhardtii* than BP-1 in terms of cell proliferation. Similarly, BP-3 was reported to be more toxic to invertebrates (i.e., *Dugesia japonica* and *Daphnia magna*) and fish than BP-1 based on the EC_50_ and lowest-observed-adverse-effect level (LOEC) values [22,41,42]. The observed differences in toxicity may be attributed to the variations in octanol-water partition coefficient (Kow) of BPs (Table 1). With a higher Kow value, BP-3 is able to pass through the double membrane and reach the action site more easily than BP-1. As a result, the tested alga tends to uptake more BP-3 from the medium, leading to a higher toxicity caused by BP-3.

Previous studies on the toxic effects of BP-type UV filters on algae employed cell number as the test endpoint. However, changes in algal cells are generally accompanied with fluctuations in photosynthetic pigments, which is indicative of photosynthesis activity and should therefore be taken into consideration as well. In the present study, a consistent reduction in concentrations of three photosynthetic pigments (i.e., chl-a, chl-b and carotenoid) was observed (Figure 2, Figure 3 and Figure 4). This indicates that BPs have a detrimental effect on photosynthetic organisms, which agrees with a previous observation [21]. Together with the reduced specific growth rate, it is speculated that the mixture of BP-1 and BP-3 affect the growth of tested alga by regulating the photosynthetic process. This is reasonable because phytoplankton algae obtain energy through photosynthesis, which converts light energy to chemical energy. Light captured in algae is absorbed directly by chl-a or by other accessory pigments (i.e., chl-b and carotenoid). Among the three pigments, chl-a was the most popular one since it is the primary photosynthetic pigment in algae and thus attracted much attention. The simultaneous reduction in chl-a content and specific growth rate agrees well with previous reports that chl-a content was positively correlated with algal biomass/cell number [43,44,45]. For example, it was reported that the effects of bisphenol A (BPA) on chl-a concentration and cell number were similar [43]. It is widely accepted that trace organic contaminants can trigger adverse effects on green algae, partially through organelles such as chloroplast [15]. The underlying mechanisms may be associated with interruption in electron transportation, phosphorylation, and production of related proteins [46]. In particular, the disruption in electron transport chain in photosynthetic processes may lead to a leakage of electrons. This may promote the formation of excited chlorophyll molecules, which can further induce the production of reactive oxygen species [47]. The oxidative stress caused by reactive oxygen species may be responsible for the reduced production of pigments [48].

Among the three pigments, carotenoids seemed to be the most sensitive (reduction rate: 15–71%). Carotenoids not only aid chl-a in harvesting light energy, but are also involved in protecting the cell (as a non-enzymatic antioxidant) from destroying by external or internal stresses. The loss of the antioxidant defense function may be a reason for the subsequent reduced concentration of chl-a and chl-b. Ding et al. (2017) reported that both chl-a and carotenoid contents in *Scenedesmus quadricauda* were reduced after exposure to 100 µg L^−1^ of naproxen [49]. Similarly, hindered growth and reduced carotenoid content were observed for green algae after exposure to nanoparticles [50,51]. These findings are consistent with our results and suggest that chloroplast is often the target of various environmental stresses.

As aquatic organisms are often exposed to mixture of BPs, possible interactive effects of two tested BPs were usually expected. The same mode of action is usually assumed for compounds in the same category [52]. Our results showed that the decreased growth rate and pigments concentrations were positively correlated with the interactive effects of the two BPs (see *a*_12_ in Table 3). To be specific, our results (positive *a*_12_ values) indicated that the two BPs have synergistic effects on the four tested parameters (i.e., growth rate, chl-a, chl-b, and carotenoid) in the studied concentration range. Synergistic effect has been reported for several UV filters. For example, in a recombinant yeast assay with the human estrogen receptor α (hERα), synergistic effects were found for a mixture of UV filters [38]. Another study also indicated a synergistic interaction between BP-3 with 4-methylbenzylidene camphor (4MBC), on the expression of heat shock protein 70 gene (*hsp70*) in *Chironomus riparius* larvae [53]. It is noteworthy to point out that the synergistic effect observed in the present study may only happen within a certain range of concentration as some studies have indicated that the interaction between UV filters may depend on the combination of concentrations [22]. 

## 5. Conclusions

The present study investigated the joint toxicity of BP-1 and BP-3 on the green alga, *C. reinhardtii*, with RSM. Photosynthetic pigments were found to be negatively affected by the two BPs, which leads to a reduction in cell growth. The cellular responses could be well illustrated by the response surface plots, contour plots and the second order polynomial regression models. These results further suggested the suitability of applying RSM in toxicity tests. To expand the application of RSM in toxicity tests, more studies are required to employ more species and contaminants.

## Figures and Tables

**Table 1:** Structure and some physicochemical properties of benzophenone-1 (BP-1) and benzophenone-3 (BP-3).

Properties	BP-1	BP-3
Synonym	2,4-Dihydroxybenzophenone	2-Hydroxy-4-methoxybenzophenone
CAS. number	131-56-6	131-57-7
Chemical structure	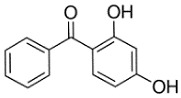	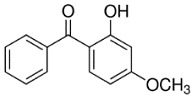
Molecular formula	C_13_H_10_O_3_	C_14_H_12_O_3_
Molecular weight (g mol^−1^)	214.22	228.24
Water solubility (mg L^−1^)	413.4	2295.40
logKow	2.96	3.79

Notes: values for boiling point, water solubility, octanol-water partition coefficient (logKow) and surface water half-life were obtained by Estimation Programs Interface (EPI) Suite developed by US EPA and Syracuse Research Corp. (SRC).

**Table 2:** Process variables used in the central composition design (CCD) model showing the coded and actual exposure concentrations of BP-1 and BP-3.

Treatment	Coded Levels		Actual Levels (mg L^−1^) ^a^	
	BP-1	BP-3	BP-1	BP-3
1	1	−1	5.0 (5.6 ± 0.3)	1.0 (0.9 ± 0.04)
2	−1	1	1.0 (0.9 ± 0.04)	5.0 (4.4 ± 0.2)
3	1.41	0	5.8 (5.7 ± 0.3)	3.0 (3.6 ± 0.2)
4	0	1.41	3.0 (2.7 ± 0.2)	5.8 (5.4 ± 0.3)
5	1	1	5.0 (4.5 ± 0.2)	5.0 (4.4 ± 0.2)
6	0	0	3.0 (2.7 ± 0.1)	3.0 (2.6 ± 0.1)
7	−1	−1	1.0 (0.8 ± 0.04)	1.0 (0.8 ± 0.04)
8	0	0	3.0 (2.6 ± 0.1)	3.0 (2.5 ± 0.1)
9	−1.41	0	0.2 (0.2 ± 0.01)	3.0 (2.6 ± 0.1)
10	0	−1.41	3.0 (2.6 ± 0.1)	0.2 (0.2 ± 0.01)

^a^ Data outside the brackets are the exposure concentrations calculated by the CCD model. Data in the brackets are the actual exposed concentrations (mean ± standard deviation) measured by LC-MS/MS.

**Table 3:** Obtained coefficients of fitted models and ANOVA for the experiments.

Term	Response			
	Specific Growth Rate	Chl-a	Chl-b	Carotenoid
Statistics for the fitted models				
*p* value	0.013	0.002	0.002	0.003
*R*^2^	94.4%	97.8%	98%	97.6%
*F* value	13.41	35.45	38.59	31.82
Lack of fit	0.0501	0.150	0.052	0.058
Statistics for the individual coefficients				
$a_{0}$	−29.95 (5.22)	−44.90 (2.80)	−32.28 (3.03)	−47.56 (3.25)
$a_{1}$	−6.23 (2.61)	−7.76 (1.40)	−5.93 (1.52)	−9.21 (1.63)
$a_{2}$	−19.88 (2.62)	−15.64 (1.40)	−19.53 (1.52)	−17.09 (1.63)
$a_{12}$	2.34 (3.69)	1.54 (1.98)	3.36 (2.14)	1.46 (2.30)
$a_{11}$	−5.98 (3.45)	−3.73 (1.85)	−4.22 (2.00)	−3.82 (2.15)
$a_{22}$	−2.08 (3.45)	5.27 (1.85)	2.09 (2.00)	5.44 (2.15)

The values highlighted in bold indicate significance (*p* < 0.05) with 95% confidence level.

## Supplementary Materials

The following are available online at http://www.mdpi.com/2305-6304/6/1/8/s1, Figure S1: A typical graph obtained by RSM, Figure S2: Dose-response curves of growth inhibition after exposing the green alga, *C. reinhardtii*, to individual BP-1 and BP-3 for 72 h. Means and standard deviations are shown. The table in the figure shows the EC_50_ values calculated for BP-1 and BP-3.

Supplementary File 1
