# Supplementary material for: Evaluating the Joint Toxicity of Two Benzophenone-Type UV Filters on the Green Alga Chlamydomonas reinhardtii with Response Surface Methodology
Source: Toxics. 2018 Jan 10;6(1):8. doi: 10.3390/toxics6010008 (PMC5874781; doi:10.3390/toxics6010008)
Supplement: Supplementary File 1 [file toxics-06-00008-s001.pdf]

Supplementary Materials: Evaluating the Joint Toxicity of Two Benzophenone-Type UV Filters on the Green Alga Chlamydomonas reinhardtii with Response Surface Methodology

Feijian Mao, Yiliang He and Karina Yew-Hoong Gin

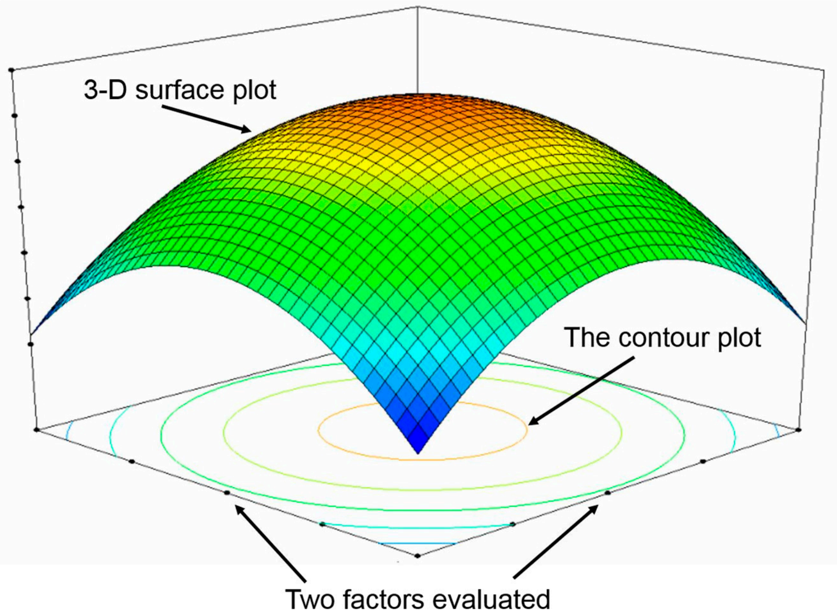

Figure S1. A typical graph obtained by RSM.

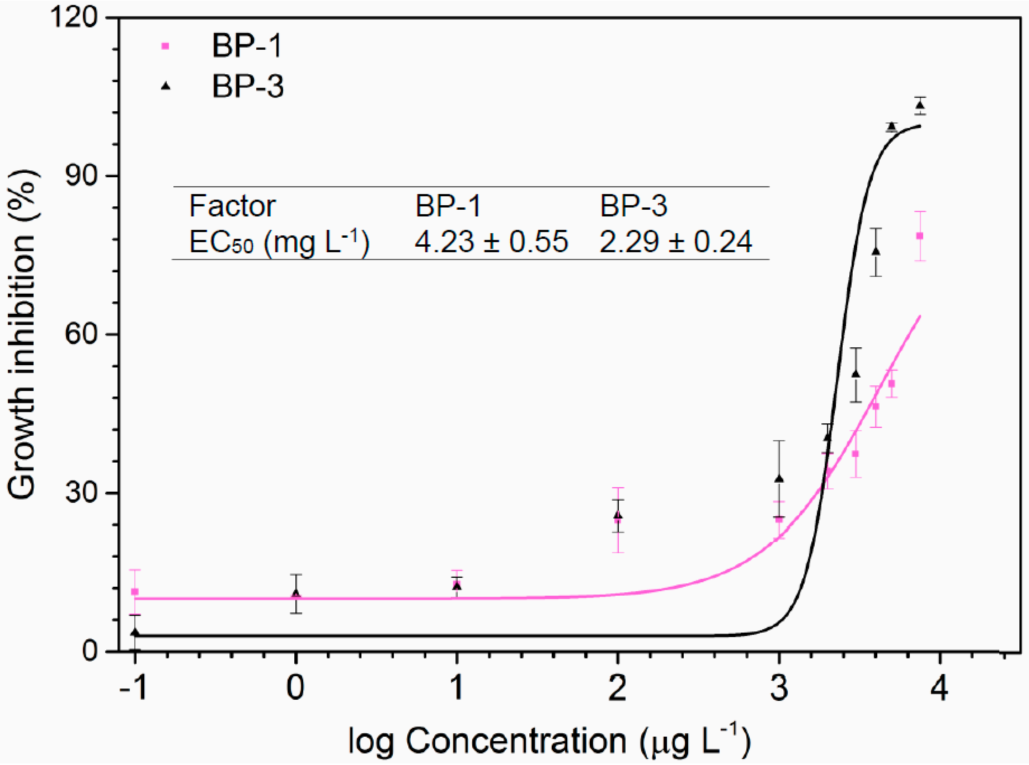

Figure S2. Dose-response curves of growth inhibition after exposing the green alga, *C. reinhardtii*, to individual BP-1 and BP-3 for 72 hours. Means and standard deviations are shown. The table in the figure shows the EC_{50} values calculated for BP-1 and BP-3.
